# Supplementary material for: Fostering Appropriate Antibiotic Use in a Complex Intervention: Mixed-Methods Process Evaluation Alongside the Cluster-Randomized Trial ARena
Source: Antibiotics (Basel). 2020 Dec 8;9(12):878. doi: 10.3390/antibiotics9120878 (PMC7764260; doi:10.3390/antibiotics9120878)
Supplement: Supplementary file 1 [file antibiotics-09-00878-s001.pdf]

Supplementary File 1:

Supplementary Table S1. Uptake of study components across groups (T2)

**Component**	**Group A** (n=68)	**Group B** (n=67)	**Group C** (n=49)	**Across Groups** (n=184)
E-learning used % (n)	84 (57)	91 (61)	86 (42)	87 (160)
QC respiratory infections used % (n)	75 (51)	88 (59)	76 (37)	80 (147)
QC urinary tract infections used % (n)	75 (51)	82 (55)	78 (38)	78 (144)
QC pneumonia used % (n)	69 (47)	76 (51)	59 (29)	69 (127)
QC multi-resistant germs used % (n)	63 (43)	73 (49)	59 (29)	66 (121)
Feedback report used % (n)	90 (61)	90 (60)	86 (42)	89 (163)
Background information used % (n)	93 (63)	87 (58)	84 (41)	88 (162)
Patient flyer German used % (n)	68 (46)	96 (64)	74 (36)	79 (146)
Patient flyer foreign language used % (n)	22 (15)	49 (33)	31 (15)	34 (63)
Website used % (n)	35 (24)	28 (19)	27 (13)	30 (56)
P4P used % (n)	66 (45)	64 (43)	37 (18)	58 (106)
Public campaign noticed % (n)	47 (32)	49 (33)	45 (22)	47 (87)
Social media noticed % (n)	6 (4)	6 (4)	8 (4)	N/A
Tablet device % (n)	N/A	33 (22)	N/A	N/A
Interdisciplinary QC % (n)	N/A	N/A	57 (28)	N/A
Computerized decision support tool % (n)	N/A	N/A	31 (15)	N/A

N/A = not applicable

Supplementary Table S2. MAs perspective on integrating study components and newly gained knowledge (T2)

**Integrating study components into practice routines was associated with great effort**	**Agree**	**Neutral**	**Disagree**
Flyer German n (%)	4 (6.9)	6 (10.3)	47 (81.0)
Flyer Foreign n (%)	6 (10.3)	7 (12.1)	38 (65.5)
Website n (%)	14 (24.1)	17 (29.3)	23 (39.7)
Social Media Content n (%)	20 (34.5)	17 (29.3)	15 (25.9)
Public campaign n (%)	17 (29.3)	18 (31.0)	17 (29.3)
Tablet device n (%)	29 (50.0)	8 (13.8)	17 (29.3)
**Transferring newly gained knowledge was associated with great effort**	Agree	Neutral	Disagree
Content of online training n (%)	9 (15.5)	9 (15.5)	38 (65.5)
Content of quality circles n (%)	13 (22.4)	16 (27.6)	27 (46.6)
Content of feedback reports n (%)	13 (22.4)	14 (24.1)	30 (51.7)
Content of background information n (%)	11 (19.0)	14 (24.1)	30 (51.7)

Supplementary Table S3. Physician perspective on new impulses provided by study components (T2)

The intervention component provided new impulses									
	**Agree**			**Neutral**			**Disagree**		
**Group**	**A**	**B**	**C**	**A**	**B**	**C**	**A**	**B**	**C**
Online Training %	48.5	59.7	40.8	25.0	19.4	18.4	22.1	16.4	34.7
Quality circles %	72.1	83.6	79.6	14.7	9.0	10.2	10.3	3.0	6.1
Feedback report %	58.8	65.7	55.1	30.9	22.4	34.7	8.8	7.5	6.1
Background information %	73.5	68.7	73.5	17.6	19.4	12.2	7.4	7.5	8.2
Patient flyer German %	35.3	53.7	53.1	17.6	28.4	24.5	39.7	14.9	20.4
Patient Flyer foreign %	13.2	19.4	22.4	8.8	26.9	26.5	64.7	40.3	40.8
Website %	14.7	14.9	20.4	16.2	19.4	18.4	61.8	52.2	51.0
Social Media %	5.9	4.5	6.1	1.5	13.4	2.0	83.8	71.6	75.5
Public campaign %	26.5	26.9	36.7	16.2	29.9	20.4	51.5	35.8	28.6
Pay for performance %	32.4	37.3	30.6	16.2	17.9	16.3	42.6	32.8	40.8
Tablet device %	N/A	9.0	N/A	N/A	19.4	N/A	N/A	61.2	N/A
Interdisciplinary quality circles %	N/A	N/A	53.1	N/A	N/A	16.3	N/A	N/A	20.4
Decision support tool %	N/A	N/A	16.3	N/A	N/A	14.3	N/A	N/A	49.0

N/A = not applicable

Supplementary File 2: Interview guide physicians (translated)

A – Use of antibiotics

Describe regular course of action when taking therapy decisions for/against antibiotics regarding patients with acute non-complicated infections

- Influence of patient preferences
- Methods or strategies in use (delayed prescribing?)

B – Effects of the quality improvement program with regard to patient care

Support of therapy decisions by provided intervention components

- Which? / Why? / Reasons / necessary changes in daily practice
- Components not used / Tailoring of components to match workflow

Influence of study participation on therapy decisions

- Changes in attitude / Changes in physician-patient conversation

C- Contextual factors – primary care network

Structure and offers in your primary care network

Role of network for daily care practice / What has changed since becoming a member?

Reasons for becoming a member

How is peer exchange organized in your network?

- Frequency of peer meetings and exchange / support for daily practice (challenges)
- Information flow / Organisation of information flow
- Information exchange regarding use of antibiotics for acute non-complicated infections

Significance of network membership regarding own course of action concerning patients with acute non-complicated infections

- Possible explanation?

D- Contextual factors – general

Further significant factors /developments regarding care for patients with acute non-complicated infections and prescribing antibiotics

- Structural conditions / Organization of processes

E- Closing

Recommendations for future use of antibiotics in patients with acute non-complicated infections

- Ideas and remarks concerning a further development of the intervention program

What would you like to tell us besides already discussed topics?

Supplementary File 3: Interview guide medical assistants (translated)

A – Antibiotic management

Please describe your range of tasks with regards to patient care in practice.

Which experiences did you make in patients with acute, uncomplicated infections?

- Standardized procedures
- Common strategies (i.e. "delayed prescribing" patterns)

B – Effects of the quality improvement program with regard to patient care

Support of therapy decisions by provided intervention components

- Which? / Why? / Reasons / necessary changes in daily practice
- Components not used / Tailoring of components to match workflow

Influence of study participation on therapy decisions

- Changes in attitude / Changes in physician-patient conversation

C- Contextual factors – primary care network

Structure and offers in your primary care network

Role of network for daily care practice / What has changed since becoming a member?

How is peer exchange organized in your network?

- Frequency of peer meetings and exchange / support for daily practice (challenges)
- Information flow / Organisation of information flow
- Information exchange regarding use of antibiotics for acute non-complicated infections

Significance of network membership regarding own course of action concerning patients with acute non-complicated infections

- Possible explanation?

D- Contextual factors – general

Further significant factors /developments regarding care for patients with acute non-complicated infections and prescribing antibiotics

- Structural conditions / Organization of processes

E- Closing

Recommendations for future use of antibiotics in patients with acute non-complicated infections

- Ideas and remarks concerning a further development of the intervention program

What would you like to tell us besides already discussed topics?

Supplementary File 4: Interview guide stakeholders (translated)

A – Warm up

Aspects of interest regarding the use of antibiotics in acute, uncomplicated infections.

- Expectations to the ARena project
- Motivation to participate

B – Influence on prescribing behavior

The rationale of the ARena project is to influence prescribing patterns in acute, uncomplicated infections. Where do you see your tasks and responsibilities regarding this topic?

- Facilitators of this rationale

C- Contextual factors – primary care network

Structure and offers in your primary care network

Role of network for daily care practice

- Arguments to join PCN
- Reasons for physicians to reject membership in PCN
- Influence of PCN membership on individual decision-making of physicians regarding care of patients with acute, uncomplicated infections

PCNs as a platform of peer exchange. How should this exchange ideally be organized?

D- Contextual factors – general

Further significant factors /developments regarding care for patients with acute non-complicated infections and prescribing antibiotics

- Structural conditions / Organization of processes, political developments

E- Closing

Recommendations for future use of antibiotics in patients with acute non-complicated infections

Strategies to sustain the goal of a rational use of antibiotics on the long-term

What would you like to tell us besides already discussed topics?
